# Thyromental Height Test as a Method for Predicting Difficult Intubation in Patients with Obesity: A Prospective Observational Study

**DOI:** 10.3390/jcm14186352

**Published:** 2025-09-09

**Authors:** Piotr Palaczyński, Jacek Smereka, Katarzyna Zawadzka-Kaczmarek, Jakub Kukliński, Hanna Misiolek, Justyna Domka, Justyna Danel, Szymon Bialka

**Affiliations:** 1 Department of Anaesthesiology and Intensive Therapy, Faculty of Medical Sciences in Zabrze, Medical University of Silesia, 40-055 Katowice, Poland; piotrpalaczynski@gmail.com (P.P.); hmisiolek@sum.edu.pl (H.M.); justynadanel@gmail.com (J.D.); sbialka@sum.edu.pl (S.B.); 2 Department of Emergency Medical Service, Wroclaw Medical University, 50-367 Wroclaw, Poland; jacek.smereka@umw.edu.pl; 3 Department of Anaesthesiology and Intensive Therapy, University Clinical Center, 80-952 Gdansk, Poland; kzawadzka@gumed.edu.pl; 4 Department of Anaesthesiology and Intensive Therapy, Faculty of Medicine, Medical University of Gdansk, 80-210 Gdansk, Poland; 5 Clinical Department of Anaesthesiology and Intensive Care, University Clinical Hospital Fryderyk Chopin in Rzeszow, 35-055 Rzeszow, Poland; justynadomka@gmail.com

**Keywords:** thyromental height test, obesity, airway management, endotracheal intubation, difficult laryngoscopy, predictor, general anesthesia

## Abstract

**Background:** Anthropometric tests and scales used to predict difficult intubation in people with obesity have limited sensitivity and specificity. A thyromental height test (TMHT) is based on the height between the anterior border of the thyroid cartilage and the anterior border of the mentum. **Objectives**: The aim of this study was to assess the usefulness of TMH in the prediction of difficult intubation in patients with obesity scheduled for elective surgical procedures. **Methods**: A prospective, observational cohort study in adult patients with BMI ≥ 30 kg⋅m^−2^ scheduled for elective surgical procedures under general anesthesia, direct laryngoscopy, and intubation was conducted in a university hospital between June 2020 and June 2021. The primary outcome measure was thyromental height, and the secondary outcome measures were thyromental distance (TMD), sternomental distance (SMD), score in the modified Mallampati test (MMT), Cormack–Lehane grade (CL), neck circumference (NC), and mouth opening (MO) distance. As a secondary outcome, a composite score was developed and analyzed for its predictive performance. **Results**: In 77 patients (56 females, 72.7%) aged 43.21 ± 9.39 years with a mean BMI of 37.18 (34.6–42.8) kg⋅m^−2^, difficult intubation was found in 18 patients (23.38%). Sleep apnea was present in 14 (23.75) patients with easy intubation vs. 9 (50%) patients with difficult intubation (*p* = 0.033). There were no statistically significant differences in thyromental height test, thyromental distance, neck circumference, and mouth opening scores. Male sex, TMD ≤ 175 mm, and MO ≤ 60.5 mm were predictors of difficult laryngoscopy. The OPERA Score (range 0–5) demonstrated superior predictive value (AUC = 0.8 *p* < 0.01), outperforming its individual components. **Conclusions**: Male sex, TMD ≤ 175 mm, and MO ≤ 60.5 mm are predictors for difficult laryngoscopy in patients with obesity. The results of our study indicate that TMH may not be a good predictor of difficult intubation in patients with obesity. However, when integrated into a composite score, it contributes meaningfully to a multifactorial risk assessment.

## 1. Introduction

Endotracheal intubation remains the gold standard for airway management in both operating room and emergency settings. The American Society of Anesthesiologist describes it as a situation where conventional laryngoscopy requires more than three failed attempts to secure the airway [1]. In clinical research, Cormack–Lehane grades III and IV, a requirement for adjunctive airway devices, or prolonged intubation times are often used as surrogate markers of difficult intubation [2]. Failure to secure the airway and ensure adequate ventilation can result in serious complications, including hypoxia, neurological damage, and cardiovascular failure. Failed intubation and severe hypoxia are recognized contributors to anesthesia-related morbidity and mortality [3,4]. In scenarios where intubation, ventilation, or oxygenation cannot be achieved, surgical airway access is recommended as a life-saving measure [5].

Patients with obesity present significant challenges in airway management, particularly during bag-mask ventilation and endotracheal intubation [6]. Difficult mask ventilation is more common in this population compared to non-obese individuals, warranting special attention during preoperative planning and airway assessment [7,8].

The association between obesity and difficult intubation remains debated. Some studies suggest that obesity is not an independent risk factor, while others report a clear correlation with increased difficulty in both intubation and bag-mask ventilation. The inconsistency in findings may be attributed to limitations such as small sample sizes, retrospective designs, or the inclusion of heterogeneous patient populations. Larger studies often focus on the general surgical population rather than exclusively on patients with obesity [9]. Despite conflicting evidence, obesity is widely recognized as a challenge in perioperative airway management, largely due to anatomical alterations associated with excess adiposity. Factors such as diagnosed obstructive sleep apnea syndrome (OSAS) and increased neck circumference are also known to contribute to difficult intubation in this population [10,11].

The prevalence of severe obesity continues to rise globally and is especially high in developed countries such as the United States and those in Europe. Reported rates of difficult intubation vary widely—from 1.5% to 20% in the general population, and up to 50% in some subgroups of patients with obesity, such as those studied in Thailand [11,12].

Endotracheal intubation in patients with severe obesity is often performed during elective bariatric surgery in specialized centers with extensive experience in the surgical and perioperative care of this population [13]. However, as the number of patients with severe obesity continues to rise, airway management is increasingly being performed by anesthesiologists who may lack specific expertise in managing obesity. This underscores the importance of developing reliable preoperative assessment tools to identify potential difficulties in airway management and ventilation [14,15].

The prediction of difficult intubation relies on validated and widely recognized preoperative assessment tools, as well as intraoperative grading systems for laryngoscopic viewing [16]. However, standard airway tests show variable accuracy, even in healthy adult populations, and should be interpreted with caution. Given their limited sensitivity, there is a clear need to develop additional predictive tests with improved diagnostic performance [11].

Several anthropometric scales and tests are currently used to predict difficult intubation in obese patients. However, most of these tools demonstrate low sensitivity and specificity, limiting their clinical reliability [17]. Recent studies suggest that simple anthropometric measurements may aid in improving airway assessment and enhancing anesthesia safety in this population [18].

TMH testing is a simple anthropometric method for predicting difficult intubation. It measures the vertical distance between the anterior border of the thyroid cartilage and the anterior border of the mentum, with the patient lying supine with their mouth closed. It has shown promise and may eventually offer advantages over other commonly used airway assessment tools. The method was first proposed by Etezadi et al., who identified a cut-off value of 50 mm [19]. However, their study focused on a general surgical population. While subsequent studies have validated TMH testing [20], meta-analyses indicate that most evaluations have been conducted in general populations rather than specifically in patients with obesity [19].

This study aimed to evaluate the clinical utility of the TMHT in predicting difficult intubation in obese patients undergoing elective surgical procedures and to compare its performance with that of other commonly used predictive tests.

## 2. Materials and Methods

**Figure 1:**
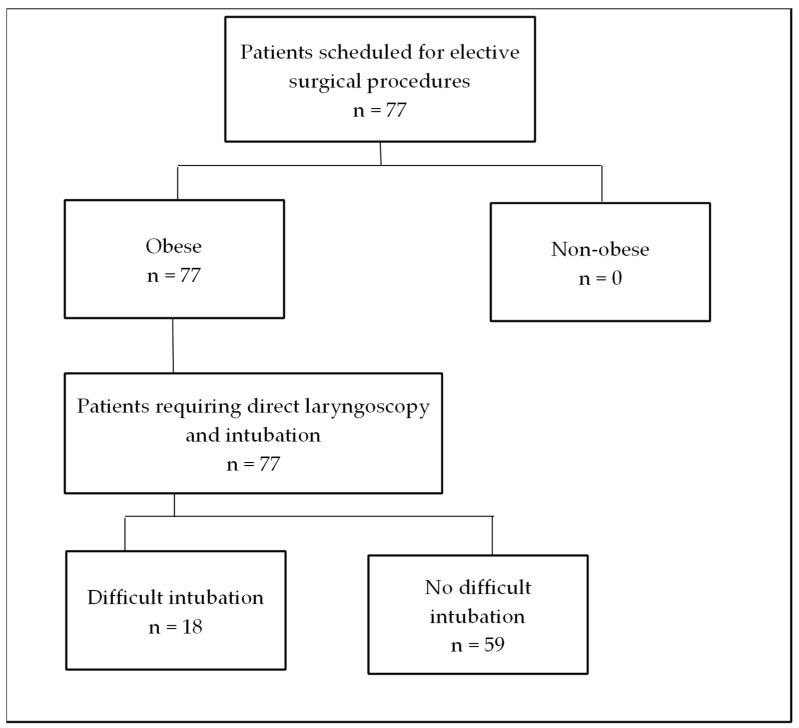
Flow chart of study.

This prospective, observational cohort study included adult patients with obesity who were scheduled for elective surgical procedures at a university hospital between June 2020 and June 2021. The first patient was enrolled on 23 September 2020. All procedures were conducted in accordance with applicable laws and institutional guidelines. The study protocol was approved by the local Bioethics Committee (approval number PCN/0022/KB1/9/20), and written informed consent was obtained from all participants. The study was registered on ClinicalTrials.gov prior to patient enrollment. The study employed convenience sampling. All patients who met the inclusion criteria were enrolled. A total of 77 patients were included, as illustrated in Figure 1.

Inclusion criteria included patients scheduled for elective surgical procedures, requiring general anesthesia, direct laryngoscopy, and endotracheal intubation, signed informed consent for participation in the trial, age over 18 years, and BMI ≥ 30 kg⋅m^−2^. 

Exclusion criteria included BMI < 30 kg⋅m^−2^, patients overweight due to ascites or tumors, emergency procedures, visible anatomic abnormalities within the head and neck, patients scheduled for awake fiberoptic intubation, intubation failure, and a lack of consent for participation in the trial. Patients meeting the exclusion criteria were withdrawn from the trial during the preoperative anesthetic visit. No data were gathered from the excluded patients.

During the routine preoperative anesthetic evaluation, the following variables were recorded: age, sex, body weight, height, ASA physical status, OSAS, and dentition status. These data were documented in Study Protocol 1 following the acquisition of written informed consent from each participant.

The primary outcome measure was TMH, and secondary outcome measures included TMD, SMD, MMT, CL, NC, and MO.

**Figure 2:**
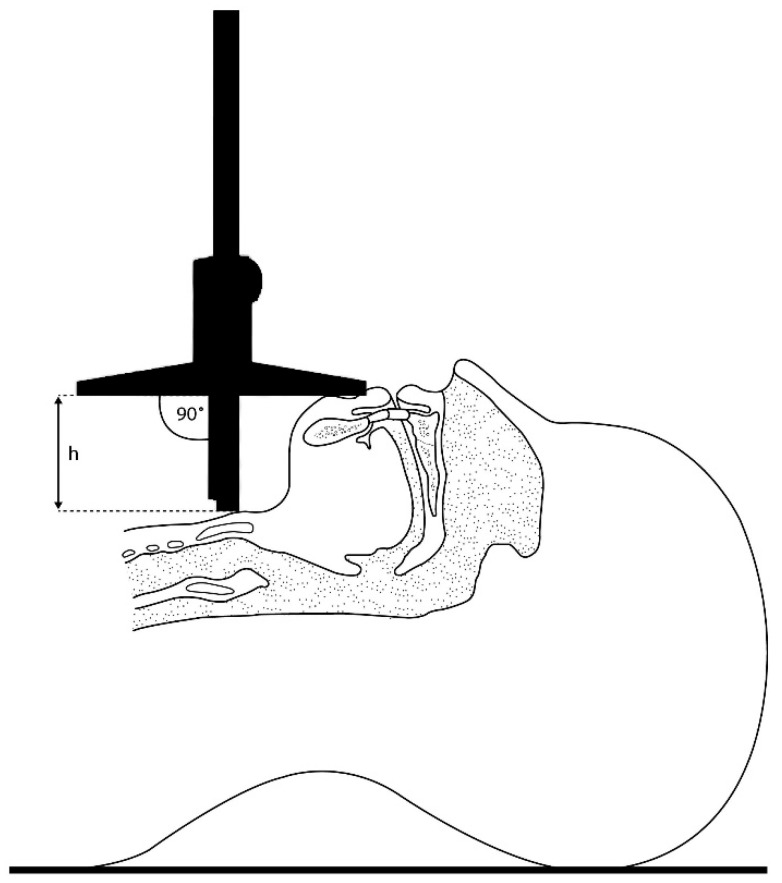
Anatomical landmarks of upper airways during TMH measurement.

**Figure 3:**
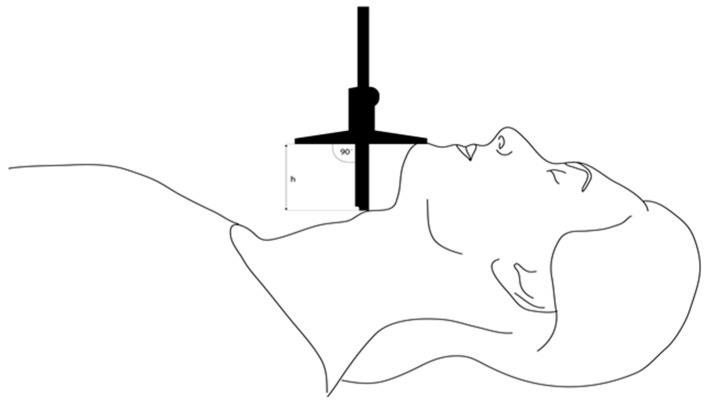
Profile view of patient positioning during TMH measurement.

**Figure 4:**
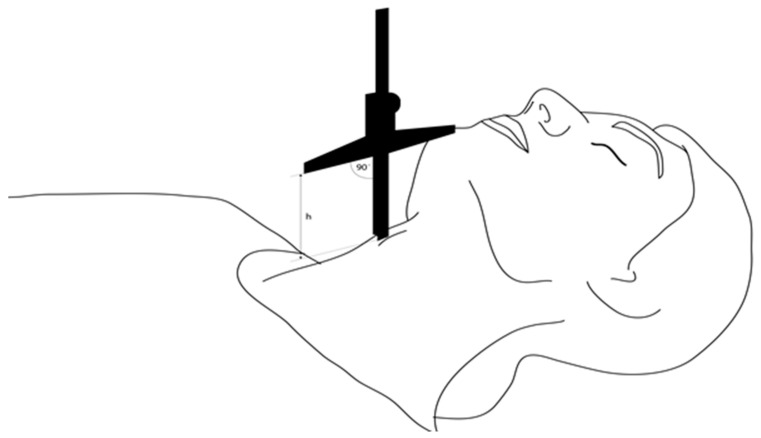
Lateral view of patient positioning during TMH measurement.

The following predictive test measurements were performed by a research team member not involved in further assessment of laryngoscopy:

- MMT: The oropharyngeal view was assessed using the modified Mallampati classification. Patients sat with their mouths maximally opened, tongue protruding, and without phonation.
- TMD was measured between the thyroid prominence and the most anterior part of the mental prominence of the mandible using a tape measure (Standard, Hoechstmas, Sulzbach, Germany), with distance in millimeters, with the patient in a supine position, head fully extended, and mouth closed.
- SMD was measured between the superior border of the manubrium sterni and the most anterior part of the mental prominence of the mandible with a tape measure (Standard, Sulzbach, Germany), with distance in millimeters, with the patient in a supine position, head fully extended, and mouth closed.
- TMH was measured as the height between the anterior border of the thyroid cartilage (on the thyroid notch just between the second thyroid laminae) and the anterior border of the mentum (on the mental protuberance of the mandible) with a depth gauge (Limit, Alingsås, Sweden), in millimeters, with the patient in a supine position with their mouth closed, as presented in Figure 2, Figure 3 and Figure 4.
- NC was measured at the level of the cricoid cartilage horizontally with a tape measure (Standard, Sulzbach, Germany) as a circumference in millimeters, with patients in the sitting position.
- MO was measured as the distance between the lower and upper incisors with a tape measure (Standard, Sulzbach, Germany), with distance in millimeters. Patients sat with their mouths maximally opened, tongues retracted, and without phonation.

All patients were anesthetized according to a standardized protocol. Standard monitoring included electrocardiography, non-invasive blood pressure measurement, and pulse oximetry. Preoxygenation was performed with 100% oxygen via a face mask for 3 to 5 min. Anesthesia induction involved propofol at 2 mg/kg of adjusted body weight and fentanyl at 2 μg/kg of ideal body weight. Neuromuscular blockade was achieved with cisatracurium at 0.15 mg/kg of ideal body weight. The depth of neuromuscular blockade was assessed using the Train-of-Four (TOF) method, and the first laryngoscopy attempt was initiated at TOF 0. Patients were positioned in either the sniffing or ramped position, and laryngoscopy was performed using a Macintosh blade size #3 or #4. Successful intubation was confirmed by bilateral chest auscultation and capnography.

All intubation attempts were performed in the operating room by an anesthesiologist who was a member of the study team and had at least one year of experience in managing airways in patients with obesity. The best laryngoscopic view was recorded using the Cormack–Lehane grading system.

Intubation was classified as “difficult” if any of the following criteria were met:

- More than two attempts were required to achieve successful intubation using a conventional Macintosh laryngoscope.
- Two failed intubation attempts were made by two experienced anesthesiologists.
- A change in technique was necessary (e.g., the use of an alternative airway device, different laryngoscope, or bougie stylet).
- Total duration of intubation attempts exceeded three minutes.

An intubation attempt was defined as the introduction of the tracheal tube past the patient’s teeth or a failed laryngoscopy attempt that did not involve tube insertion.

Prior to the start of the trial, all members of the research team underwent training to ensure standardized measurement techniques. The accuracy of data entry was monitored monthly by comparing entries in the electronic database with the Study Log.

Data collected during the preoperative visit by a research team member were recorded in Study Protocol 1. Intraoperative data were documented by the anesthesiologist performing the procedure in Study Protocol 2. All data were transcribed daily into an electronic database by a designated member of the research team.

All enrolled patients were recorded in the Study Log, along with any observed deviations, potential sources of bias, or procedural errors. Study Protocol 2 specifically included documentation of the best laryngoscopic view based on the Cormack–Lehane grading system and the presence or absence of difficult intubation.

Since no intervention or allocation to different treatment arms was performed, the inclusion of the entire eligible population provided a representation of the target group without the need for randomization. To minimize bias, anesthesiologists responsible for laryngoscopy and intubation were blinded to the preoperative measurements, which were obtained by separate members of the research team. 

As part of the secondary analysis, we developed an exploratory index, the OPERA Score (Obesity Parameters for Evaluating Risk in Airway management), designed to assist in predicting difficult intubation specifically in patients with obesity. The score was constructed based on five simple clinical and anatomical predictors available preoperatively: thyromental height (TMHT < 50 mm), Mallampati score (class III–IV), neck circumference (≥430 mm), mouth opening (<35 mm), and history of obstructive sleep apnea (OSA). Each variable contributed 1 point to the score, resulting in a range from 0 to 5 points. To assess its diagnostic performance, the OPERA Score was compared to its individual components using ROC analysis. Logistic regression was performed to explore the relationship between increasing score values and the likelihood of difficult intubation. To evaluate the predictive utility of the OPERA Score, we treated it as a composite point-based clinical test with integer values ranging from 0 to 5. This approach aligns with the intended clinical use of the tool as a simple, bedside risk stratification instrument. The discriminative performance of the score was assessed using receiver operating characteristic (ROC) curve analysis, with the area under the curve (AUC) serving as the primary measure of accuracy. Given that the OPERA Score is a variable with limited resolution and a fixed scale, it was analyzed as a discrete classifier rather than a continuous predictor. While we also performed bootstrap-based confidence interval estimation for comparison, the limited sample size, unequal distribution of score categories, and small number of difficult intubation cases resulted in unstable intervals with wide bounds.

### Statistical Analysis

The data distributions were analyzed by using the Shapiro–Wilk normality test. Categorical variables were presented as the number of observations and percentages, with continuous variables as the mean, standard deviation (±SD), median, and interquartile range (Q1–Q3). The Mann–Whitney U test was applied for two-group comparison. Frequency analysis was conducted with Fisher’s exact test or the chi-square test. All calculated *p* values were two-sided, and *p* ≤ 0.05 was considered significant.

The stepwise multivariable logistic regression method was used to determine independent predictors of difficult laryngoscopy. *p* > 0.1 was an exclusion criterion in the univariable analysis. For logistic regression analysis, the variables “TMH”, “TMD”, and “MO” were dichotomized, and cut-off points were calculated by trees (CART) analysis. Odds ratios and 95% confidence intervals (±95%CI) were also calculated.

The diagnostic potential of selected variables was determined by receiver operating characteristic (ROC) analysis and expressed in terms of the area under the ROC curve (AUC). Accuracy, sensitivity, specificity, and standard error (SE) were also calculated. Statistical analyses were performed using STATISTICA 13.3 software (Tibco Software Inc., Palo Alto, CA, USA).

## 3. Results

Basic demographic and clinical data are presented in Table 1. 

Of the 77 patients included in the study, difficult intubation occurred in 18 patients (23.4%). There were no statistically significant differences between the easy and difficult intubation groups in terms of demographic data. OSAS was present in 14 patients (23.7%) in the easy intubation group and in 9 patients (50%) in the difficult intubation group, a difference that reached statistical significance (*p* = 0.033). There were no significant differences between groups in TMH, TMD, NC, or MO values. However, SMD was significantly shorter in the difficult intubation group (median 170.0 mm) compared to the easy intubation group (median 180.0 mm, *p* = 0.03). Further details regarding measurements are presented in Table 2. 

Intubation time was significantly longer in the group with difficult intubation (29.5 s vs. 13.0 s, *p* < 0.0001). The number of intubation attempts was higher in the difficult intubation group (*p* = 0.0013), and the use of the bougie stylet was 27.8% vs. 0% (*p* < 0.0001). Modification of intubation/laryngoscopy technique or bougie use was necessary for eight patients (44.4%) in the difficult intubation group vs. zero in the easy intubation group (*p* < 0.0001). Differences in demographic, anthropocentric, and anesthesia induction parameters between the patients with easy and difficult laryngoscopy are presented in Table 2.

Detailed comparisons of demographic characteristics, anthropometric measurements, and anesthesia induction parameters between patients with easy and difficult laryngoscopy are presented in Table 2.

In eight patients, modification of the intubation or laryngoscopy technique, including the use of a bougie, was required. Within this subgroup, there were no statistically significant differences compared to the group that did not require technique modification or bougie use in terms of age, BMI, ASA classification, dentition status, MMT, OSAS, TMH, TMD, SMD or MO. However, patients who required modified techniques or bougie use had significantly greater NC and a higher neck circumference-to-TMH ratio (9.00 vs. 7.20, *p* = 0.037) compared to those who did not require such modifications. Detailed comparisons of demographic, anthropometric, and anesthesia-related parameters between these two groups are presented in Table 3.

**Figure 5:**
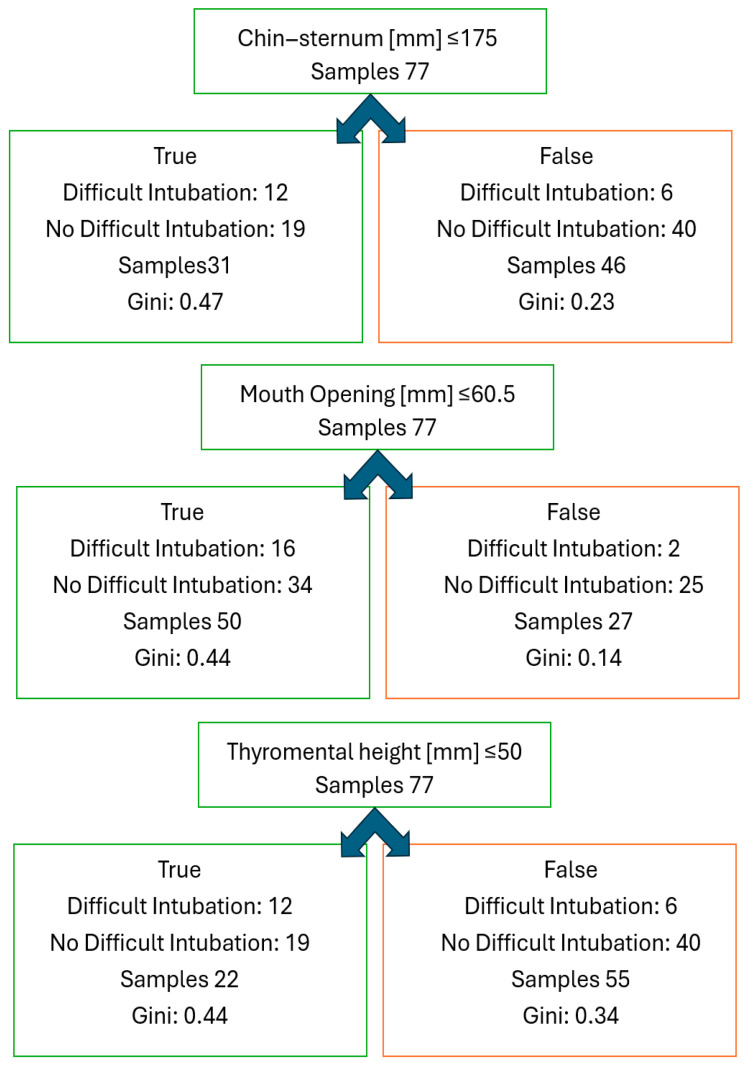
CART model for prediction of difficult laryngoscopy; independent variables: chin–sternum distance, thyromental height, and mouth opening.

A classification tree analysis (CART) was performed to identify the most informative predictors of difficult intubation among the study population (Figure 5). The tree model evaluated three variables: SMD, MO, and TMH.

Multivariable logistic regression analysis identified three independent predictors of difficult laryngoscopy: male sex, SMD < 175 mm, and MO < 60.5 mm. Detailed results of the regression model are presented in Table 4 and Table 5.

TMH was evaluated as the primary predictor of difficult airway intubation. Based on the applied threshold, it demonstrated a sensitivity of 33.3% (95% CI: 13.3–59.0%) and a specificity of 72.9% (95% CI: 59.7–83.6%). These results indicate that TMH correctly identified one-third of difficult intubations, while maintaining moderate ability to exclude those with easy airway intubation.

The positive predictive value (PPV) was 27.3% (95% CI: 10.7–50.2%), reflecting a relatively low probability that a positive TMH result corresponds to a true difficult intubation. In contrast, the negative predictive value (NPV) was 78.2% (95% CI: 65.0–88.2%), suggesting that a negative TMH result was more likely to correctly predict easy airway intubation. The overall accuracy of TMH was calculated at 63.6% (95% CI: 51.9–74.3%), indicating moderate diagnostic performance.

The proposed OPERA Score was associated with increasing likelihood of difficult intubation. When assessed as a simple classifier, the OPERA Score demonstrated discriminatory performance in predicting difficult intubation, with an AUC of 0.80. This result reflects its intended use as a clinical screening tool for bedside risk stratification, where risk clearly increased with higher point totals (6.5% at 0 points vs. 80.1% at 5 points). However, when the same score was entered into a logistic regression model and evaluated using cross-validated predicted probabilities, the AUC was lower (approximately 0.71). This discrepancy highlights the distinction between a categorical risk score used for clinical decision-making and its treatment as a continuous variable in a statistical model. Therefore, the AUC of 0.80 should be interpreted as a diagnostic performance metric of the OPERA Score as a structured ordinal test, not as a regression-based predictor. Additional external validation in wider studies is needed to confirm its generalizability. Importantly, we tested the impact of each component on the overall performance of the OPERA Score. Exclusion of the TMHT from the model led to a reduction in the AUC from 0.8 to 0.64, and the resulting composite no longer reached statistical significance (*p* > 0.05). Although the TMHT did not achieve statistical significance on its own, its integration within the OPERA Score proved essential to preserving the model’s predictive strength. This finding highlights the importance of combining weaker individual predictors into a composite measure that reflects anatomical and functional airway complexity (Table 6).

**Figure 6:**
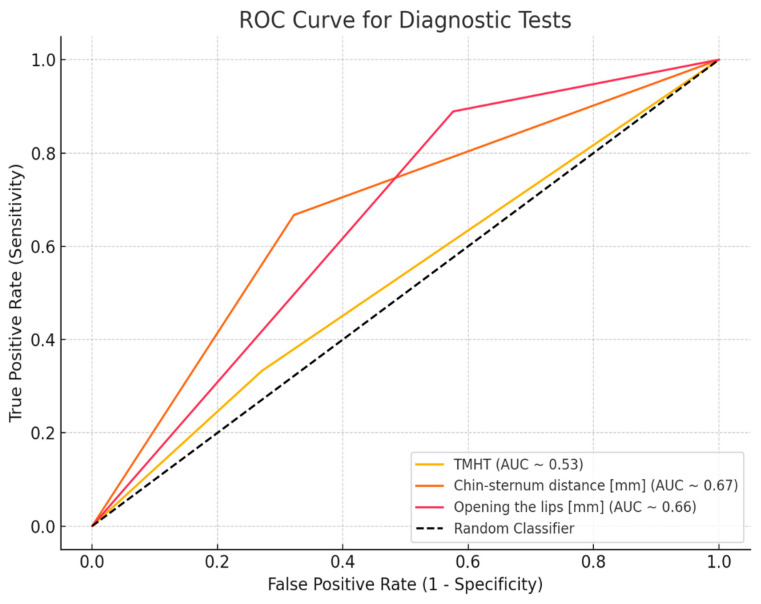
Comparison of ROC curves for “thyromental height”, “chin–sternum distance”, and “mouth opening” variables as predictors of difficult laryngoscopy.

Figure 6 compares the ROC curves for “TMH”, “SMD”, and “MO” variables as predictors of difficult laryngoscopy.

## 4. Discussion

Our study found that difficult intubation occurred in approximately 23% of obese patients undergoing elective surgery. Several demographic and clinical parameters—including age, sex, BMI, ASA classification, dentition status, MMT, TMD, TMH, SMD, NC, and MO, did not significantly differ between the easy and difficult intubation groups.

The primary aim of this study was to assess the utility of the thyromental height test as a predictor of difficult intubation in patients with obesity. TMH values did not differ significantly between groups, indicating limited diagnostic utility in this population [21]. Although TMH testing demonstrated moderate specificity, its low sensitivity and positive predictive value suggest that it is insufficient as a standalone tool for preoperative airway assessment in obese patients.

When evaluating difficult endotracheal intubation, it is important to distinguish between challenges related to visualizing the airways and those related to advancing the endotracheal tube through the airway [17]. In patients with obesity, both components may be impaired due to anatomical and physiological changes [6,18]. Factors such as a high BMI, increased NC, and OSAS are frequently associated with difficulty in both visualization and tube insertion [22].

The role of OSAS as a predictor of difficult intubation has been widely documented [23]. In our study, the presence of OSAS and reduced SMD were the only variables that significantly differed between the easy and difficult intubation groups. Furthermore, multivariable logistic regression analysis identified male sex, SMD < 175 mm, and MO < 60.5 mm as independent predictors of difficult laryngoscopy.

In the subgroup of patients who required modification of the intubation or laryngoscopy technique, including bougie use, both NC and the NC-to-TMH ratio were significantly higher compared to in those who did not require such modifications (9.00 vs. 7.20; *p* = 0.037). Similarly, Kim et al. reported that the ratio of NC to TMD was a more accurate predictor of difficult intubation than many traditional bedside tests [24]. In our study, patients in the modification group also had significantly greater neck circumference (450.0 mm vs. 400.0 mm, *p* = 0.011), further supporting the relevance of this parameter.

TMH has been evaluated in multiple studies involving surgical patients, including those with obesity. However, variations in study populations, degrees of obesity, and methodological differences may explain the inconsistent results observed across the literature. Moreover, many of these studies included relatively small sample sizes, which may limit the generalizability of their findings.

Panjiar et al. evaluated the utility of TMH in a cohort of 550 adult patients undergoing elective surgery under general anesthesia and concluded that TMH testing had the highest accuracy among the predictive tests assessed [25]. Similar findings were reported by Rao et al. [26], Etezadi et al. [19], and Jain et al. [27], the latter focusing on patients undergoing cardiac surgery. While these studies support the potential value of TMH, they primarily involved general adult populations and did not specifically target patients with obesity. The authors of these studies also emphasized the need for further research in more diverse and specific patient populations.

Mostafa et al. examined the accuracy of TMH in predicting difficult intubation among 120 elderly patients scheduled for elective procedures and found that both TMH testing and the MMT had fair predictive value, while TMD and SMD performed poorly [28]. Similarly, Rawal et al. [29] and Prakash et al. [30] reported moderate predictive ability of TMH in general patient populations. However, they noted a high rate of false positives and relatively low overall accuracy, concluding that TMH should not be used as a standalone tool for predicting difficult laryngoscopy. These conclusions were further supported in studies involving double-lumen tube intubation, although those investigations also did not focus on obese patient groups [29].

One of the few studies that did not support the effectiveness of the TMH was conducted in Japan by Yabuki et al. [31]. In a meta-analysis by Carvalho et al., eight studies involving a total of 2844 patients were evaluated. Although this analysis was not specific to patients with obesity and included a general adult population, it revealed significant heterogeneity across the included studies. Nonetheless, the results suggested that TMH testing is a fair predictor of difficult laryngoscopy in mixed adult populations and performed better than most other bedside airway assessment tests [21].

Similarly, a more recent meta-analysis by Chen et al. examined the diagnostic accuracy of various preoperative tests used to predict difficult intubation in adult patients undergoing surgery. This analysis also confirmed the utility of TMH testing in identifying difficult intubation under direct laryngoscopy, reporting that it outperformed several other conventional preoperative assessments [32]. However, as with Carvalho et al., this meta-analysis did not focus on obese patient populations. Furthermore, significant heterogeneity among the included studies—particularly regarding the application of external laryngeal manipulations—limits the generalizability of these findings to specific subgroups such as patients with obesity.

In a large retrospective study conducted in the United States, Moon et al. analyzed all endotracheal intubations performed in the operating room over a six-year period, encompassing 45,447 cases. Of these, 1893 cases (4.2%) were classified as difficult intubations. Interestingly, the incidence of difficult intubation was not higher among patients with severe obesity compared to those without. However, patients with severe obesity demonstrated a significantly greater incidence of difficult mask ventilation. Predictive factors for both difficult mask ventilation and difficult intubation included age over 46 years, male sex, and an MMT of 3 or 4 [8].

Magalhães et al. examined the relationship between clinical predictors and the incidence of difficult face mask ventilation and laryngoscopy in 88 patients with obesity undergoing surgery under general anesthesia. Their findings showed that the presence of OSAS strongly correlated with difficult laryngoscopy. Comparisons between obese and non-obese patients revealed significant differences in key airway parameters, including TMD (81 ± 14 mm vs. 76 ± 10 mm), NC (407 ± 34 mm vs. 364 ± 40 mm), and BMI (36.7 ± 6.1 kg/m^2^ vs. 24.7 ± 3.1 kg/m^2^). Notably, the average BMI in the Magalhães et al. study was lower than that of our study population [33].

There are relatively few studies specifically evaluating the use of TMH testing in the preoperative assessment of patients with obesity. Siriussawakul et al. examined predictive models in a Thai population of obese patients and found that these models provided only moderate benefit in identifying intubation difficulties during direct laryngoscopy [34]. The existing literature on predictive airway assessment tools in this population remains heterogeneous, with inconsistent results across studies [32]. Some reports have suggested the utility of individual parameters such as increased neck circumference, TMH, or the presence of OSAS [33,35,36,37,38]. However, similar assessments in pregnant women with obesity undergoing cesarean section have also shown limited predictive value for these commonly used scales [39].

Ahmed et al. specifically investigated the predictive value of TMH testing in patients with BMI > 30 kg/m^2^ undergoing elective surgery with direct laryngoscopy and endotracheal intubation [40]. In their study, the mean BMI was 43.7 ± 6.6 kg/m^2^, notably higher than that of our cohort (mean BMI: 37.18 kg/m^2^; range: 34.6–42.8). Their multivariate analysis identified only the MMT and TMHT as independent predictors of difficult intubation. Importantly, TMH showed good predictive performance when a lower cut-off value of <47 mm was applied, suggesting that the threshold used may significantly influence its diagnostic accuracy.

Selvi et al. proposed alternative anthropometric measurements, including chin–nape circumference and the ratio of neck circumference to chin–nape circumference, as potential predictors of difficult mask ventilation and intubation in obese patients. Their study underlines the growing interest in morphometric parameters beyond traditional airway tests, particularly those reflecting the anatomical alterations associated with obesity. In this context, our results provide additional evidence that simple, bedside measurements such as the TMH test could serve as practical tools in preoperative assessment, complementing more complex anthropometric indices described in the recent literature [41]. 

Kheirabadi et al. evaluated multiple clinical predictors for difficult endotracheal intubation in patients with obesity. Among the tests assessed, the TMH test and the Upper Lip Bite Test (ULBT) demonstrated the highest sensitivity for predicting difficult intubation under direct laryngoscopy [42].

Sharma et al. evaluated several commonly used indices for predicting difficult airway management in obese patients and highlighted the limitations of relying on a single parameter. Their finding demonstrated that although individual tests possess some predictive value, their sensitivity and specificity remain suboptimal when applied in isolation. Our results complement those of Sharma et al. by emphasizing the need for reliable, practical, and reproductible indices tailored to obese populations, in whom conventional predictors frequently underpower [43]. 

The challenge of accurately predicting difficult intubation remains a central focus in airway management research. The development of reliable, simple, and bedside-accessible screening tools is critical for improving perioperative safety. While TMH testing does not appear to be the definitive “gold standard” for predicting difficult airway intubation in patients with obesity, a number of studies have demonstrated its potential clinical utility, particularly in general patient populations [19,20,34].

Our study has several limitations. The primary limitation is the relatively small sample size, which may have affected the statistical power and generalizability of the findings. Additionally, the range of BMI values in our study population was relatively narrow, and the proportion of patients with an extremely high BMI was low. This may limit the applicability of our results to populations with morbid or super obesity. Furthermore, this was a single-center study conducted in a non-diverse population, which may also affect the external validity of our conclusions. 

A formal power analysis was conducted prior to the study; however, due to population-related constraints, it was not possible to recruit a sufficiently large study group. As a result, with a total sample size of 77 and 18 cases of difficult intubation (23%), the study was underpowered to detect moderate effect sizes with adequate statistical confidence. This limitation may affect the generalizability and robustness of our findings. Furthermore, we acknowledge that the observed incidence of difficult intubation (23%) is higher than that reported in some previous studies for obese patients with BMI < 50 kg/m^2^ [15,40,42,44,45,46,47], which should be interpreted in light of our specific patient population and setting.

Continuous variables such as MO and SMD were dichotomized based on cut-off values derived from CART analysis. While this approach facilitates clinical interpretation, we acknowledge that dichotomization may lead to information loss and potential bias. The selected thresholds (e.g., MO < 60.5 mm) were determined from data-driven recursive partitioning rather than predefined clinical guidelines. We recognize this as a limitation and suggest that future studies explore alternative modeling strategies that preserve continuous variable structures to enhance predictive performance.

Stepwise logistic regression was employed for variable selection, which is a common but sometimes criticized approach due to the risk of overfitting and model instability. The current analysis did not include internal validation techniques such as cross-validation or bootstrapping, nor was an independent dataset used for external validation. These aspects should be addressed in future research to confirm the stability and generalizability of the proposed predictive model.

In constructing the multivariable model, variables were initially screened using univariable logistic regression. Only those with *p*-values < 0.1 were considered for inclusion in the stepwise procedure. We acknowledge that this approach may omit clinically relevant confounders that do not show univariable significance. Future studies should consider forced inclusion of such variables based on clinical judgment rather than purely statistical criteria.

In this study, airway assessment tests most frequently utilized in the participating institution and routinely applied in daily practice were selected. It is recognized that not all potential predictors of difficult intubation, including the Upper Lip Bite Test, were assessed. Given that airway measurements were performed manually, inter-observer variability may influence measurement reliability. Although all examiners were trained anesthesiologists following standardized protocols, no formal assessment of inter-observer agreement (e.g., intraclass correlation coefficient) was conducted. This represents a limitation that should be addressed in future studies.

The area under the curve (AUC) values for individual predictors remained below 0.7, indicating limited discriminatory performance. This limitation suggests that while the variables studied may have some association with difficult intubation, their standalone predictive utility in clinical settings is constrained. A more comprehensive model incorporating additional features and external validation is warranted.

What is more, an excessive amount of adipose tissue may affect the identification of anatomical landmarks of the head and neck, which is crucial for correct measurement of anthropometric tests and scales used to predict difficult intubation, such as TMH testing. Recent studies emphasize the use of ultrasound imaging to enhance the performance of such tools [48,49]. We believe that further research into the prediction of difficult intubation in patients with obesity, including TMH testing, may benefit from relying on the utilization of ultrasound as an assistance to anthropometric measurements. 

As small differences in the measurements of presented parameters may influence the prediction of difficult intubation, it is crucial to properly carry out the examination. Conditions such as the degree of neck extension, mouth closure, muscle relaxation, and surface and inclination of the bed may all greatly influence the results of measurements. We see this as a possible limitation of our study as TMH testing among other parameters is sought to be easily obtainable and replicable. Different clinical scenarios such as time-sensitive emergency procedures or patients with dyspnea who may not tolerate the supine position may prove even more challenging in acquiring reliable results.

Among potential parameters that may predict difficult intubation, selected few were chosen in our study: MMT, TMD, SMD, TMHT, MO, and NC. Recent studies show that methods such as the ULBT may be even more successful in predicting difficult airway intubation [3]. Non-inclusion of such predictors in the presented analysis is a limitation of our study. The method of obtaining MO measurements was carried out using measuring tape in contrast to other studies where calipers were used. Such a decision allowed for a more unified method of measurement with other studied parameters, but at the cost of precision and accuracy. We see the possible underperformance of measuring tape as a limitation of our study. Other limitations can be found in the use of the four-step Cormack–Lehane grading scale, instead of the modified, more detailed, and more recent six-step classification. Also, what stands out in the results of our study is the high prevalence of patients with TMD lower than 60mm, which is an independent predictor of difficult intubation. Correlation can be found in the overall high level of difficult intubation observed in the examined population. 

Although TMH alone showed only modest predictive value, its inclusion within the OPERA Score enhanced overall model performance. This highlights a key insight: individual predictors may be limited in isolation, but when integrated into a composite score, they can meaningfully contribute to risk stratification. The rationale for building the OPERA Score was the clinical reality that difficult intubation in obese patients is multifactorial. No single anatomical measurement captures the complexity of airway management. By combining accessible features, we aimed to reflect both anatomical constraints and functional risk. It is important to emphasize that the OPERA Score was developed within this study. It has not been validated externally and should therefore be interpreted as an exploratory tool, useful for hypothesis generation and clinical reflection, rather than a definitive clinical guideline.

The underperformance of TMH testing in the studied population may be attributed to several factors. Firstly, the excessive amount of adipose tissue may influence the proper identification of anatomical landmarks, thus undermining the correct measurement of TMH. The high prevalence of patients with a high NC in the studied population may validate this hypothesis to some extent. What is more, the distribution of adipose tissue in the head and neck region may differ among patients, which influences the prevalence of the predicted difficult airways, as studied by Akin et al. [50]. The occurrence of difficult intubation is typically attributed to a distinctive morphology of the head, neck, and airway anatomy, where many variables impact the final outcome. Previous studies show that among other parameters, NC plays an important role in this specific population [13]. It can be hypothesized that TMH may capture the distinctiveness of the anatomy in this specific population. Furthermore, proper visualization of the airways is not the only factor behind successful intubation, as the introduction of the tube into the airways may be challenging due to other circumstances. During the COVID-19 pandemic, the need for personal protective equipment (for example goggles that may hinder visibility or N95 masks) might have had impact on the prevalence of difficult intubation [51]. Other factors may include an abnormally large tongue or dentition, pharynx structure, enlarged tonsils, or laryngeal and tracheal stenosis, among many others. Our results show that the incidence of difficult intubation did not necessarily correlate with a high CL grade in every case. TMH may not be effective in capturing the factors outside of the morphology of the head and neck [52]. 

Evaluation of the thyromental height test may require further research. Although the results of studies vary, the test often appears to be very useful in the general patient population, and it can be used in the operating room setting and emergency medicine [33]. The cut-off point, set at different values in different studies, may be particularly important, making it difficult to apply this assessment in clinical practice [19]. Further randomized trials on large groups of patients are needed to evaluate the usefulness of new methods of preoperative evaluation of severely obese patients to reduce further risk of complications associated with airway management. 

What is more, several recently published studies and guidelines highlights the use of videolaryngoscopy as the gold standard of airway management in patients with obesity [15,53,54]. Further research should evaluate the usefulness of TMH in prediction of difficult airway intubation using videolaryngoscopy in selected group of patients. 

## 5. Conclusions

The findings of this study are consistent with previously reported anatomical limitations that impair visualization of the glottis and increase the likelihood of requiring alternative or adjunctive airway management strategies.

TMH, evaluated as the primary outcome, showed markedly limited diagnostic utility, indicating poor discriminatory performance. These results suggest that, in this patient group, TMH does not provide sufficient sensitivity to reliably identify difficult airways preoperatively. The poor performance of TMH testing may stem from multiple factors. First, the pathophysiology of the obese airway is complex and multifactorial; no single measurement is likely to capture all anatomical contributors to intubation difficulty. Second, the use of a single cut-off value for TMH limits the granularity of the analysis. It is possible that alternative thresholds or continuous analysis would yield more informative results. Third, inter-operator variability and measurement inconsistency may further reduce the reliability of TMH testing in everyday clinical settings.

This study points toward a multifactorial burden of difficult intubation in which no single marker is sufficient, but several combined features might offer better predictive power. Proposed in this study, the OPERA Score is an index developed to predict difficult intubation in obese patients. Comprising five simple preoperative features, it demonstrated superior predictive value compared to individual tests. While the model has not yet been externally validated, it represents a promising step toward integrated airway risk assessment and highlights the importance of multifactorial thinking in obese populations.

It is important to emphasize that our study has notable limitations, including a relatively small sample size and a limited number of difficult intubation cases. 

Despite these limitations, this study contributes valuable insight into the usefulness of TMH in obese patients and reinforces the need for multimodal, individualized airway assessment strategies. Further studies are warranted to validate these findings in larger, more diverse populations, preferably in multicenter prospective designs. The development of reliable, easy-to-perform, and objective airway screening tools remains a priority in perioperative medicine, especially in high-risk groups such as patients with obesity.

## Figures and Tables

**Table 1:** Demographic and clinical characteristics of study group (*n* = 77).

Variable		Number (%), Mean ± SD or Median (Q1–Q3)
Age (years)		43.21 ± 9.3
Gender	male	21 (27.3)
	female	56 (72.7)
BMI (kg·m^−2^)		37.18 (34.6–42.8)
ASA	I	1 (1.3)
	II	34 (44.2)
	III	42 (54.5)
Condition of the dentition	1	63 (81.8)
	2	8 (10.4)
	3	3 (3.9)
	4	3 (3.9)
OSAS	no	54 (70.1)
	yes	23 (29.9)

Descriptive data were presented as the number of observations (per cent), mean ± SD, or median (Q1–Q3). Condition of dentition: 1—full dentition, 2—partial upper dentition, 3—partial lower dentition, and 4—no dentition.

**Table 2:** Differences in demographic, anthropocentric, and anesthesia induction parameters between patients with easy and difficult intubation.

Variable		Easy Laryngoscopy (*n* = 59)	Difficult Laryngoscopy (*n* = 18)	*p*-Value
Age (years)		43.0 (34.0–48.0)	45.0 (42.0–48.0)	0.124
Gender	male	13 (22.0)	8 (44.4)	0.061 ^#^
	female	46 (78.0)	10 (55.6)	
BMI (kg·m^−2^)		38.1 (34.6–43.0)	36.4 (33.9–41.2)	0.329
ASA scale	I	1 (1.7)	0 (0.0)	0.749
	II	25 (42.4)	9 (50.0)	
	III	33 (55.9)	9 (50.0)	
Condition of dentition	1	48 (81.4)	15 (83.3)	0.621
	2	6 (10.2)	2 (11.1)	
	3	3 (5.1)	0 (0.0)	
	4	2 (3.4)	1 (5.6)	
Sleep apnea	no	45 (76.3)	9 (50.0)	0.033 *
	yes	14 (23.7)	9 (50.0)	
Thyromental height test (TMHT) [mm]		53.0 (48.0–70.0)	57.0 (45.0–66.0)	0.823
Thyromental distance (TMD) [mm]		90.0 (80.0–105.0)	90.0 (80.0–100.0)	0.805
Chin–sternum distance [mm]		180.0 (170.0–190.0)	170.0 (150.0–180.0)	0.030 *
Mallampati scale	I	18 (30.5)	3 (16.7)	0.248
	II	25 (42.4)	8 (44.4)	
	III	12 (20.3)	7 (38.9)	
	IV	4 (6.8)	0 (0.0)	
Neck circumference [mm]		400.0 (380.0–440.0)	410.0 (390.0–460.0)	0.150
Mouth opening [mm]		60.0 (50.0–70.0)	50.0 (50.0–60.0)	0.070 ^#^
Intubation time [sec]		13.0 (10.0–19.0)	29.5 (19.0–66.0)	<0.0001 *
Number of intubation attempts	1	58 (98.3)	13 (72.2)	0.0013 *
	2	1 (1.7)	4 (22.2)	
	5	0 (0.0)	1 (5.6)	
Use of bougie stylet	no	59 (100.0)	13 (72.2)	<0.0001 *
	yes	0 (0.0)	5 (27.8)	
Cormack–Lehane grade	I	50 (84.8)	0 (0.0)	<0.0001 *
	II	7 (11.9)	9 (50.0)	
	III	2 (3.4)	4 (22.2)	
	IV	0 (0.0)	5 (27.8)	
Modification of intubation technique or bougie use	no	59 (100.0)	10 (55.6)	<0.0001 *
	yes	0 (0.0)	8 (44.4)	

Descriptive data are presented as the number of observations (per cent) or median (Q1–Q3). *: statistically significant; ^#^: a tendency toward statistical significance; condition of the dentition: 1—full dentition, 2—partial upper dentition, 3—partial lower dentition, and 4—no dentition.

**Table 3:** Differences in demographic, anthropocentric, and anesthesia induction parameters between patients with or without modification of intubation/laryngoscopy technique or bougie use.

Variable		Modification-No (*n* = 69)	Modification-Yes (*n* = 8)	*p*-Value
Age (years)		43.00 (36.00–48.00)	43.50 (42.00–47.50)	0.287
Gender	male	54 (78.26)	2 (25.00)	0.004 *
	female	15 (21.74)	6 (75.00)	
BMI (kg·m^−2^)		37.10 (34.54–42.77)	40.20 (36.31–43.57)	0.300
ASA scale	I	1 (1.45)	0 (0.00)	0.462
	II	32 (46.38)	2 (25.00)	
	III	36 (52.17)	6 (75.00)	
Condition of dentition	1	57 (82.61)	6 (75.00)	0.461
	2	6 (8.70)	2 (25.00)	
	3	3 (4.35)	0 (0.00)	
	4	3 (4.35)	0 (0.00)	
OSAS	no	52 (75.36)	2 (25.00)	0.007 *
	yes	17 (24.64)	6 (75.00)	
TMH [mm]		56.00 (49.00–70.00)	47.5 (42.50–57.50)	0.163
TMD [mm]		90.00 (80.00–100.00)	85.00 (77.50–100.00)	0.713
SMD [mm]		180.00 (170.00–190.00)	170.00 (160.00–180.00)	0.118
MMT	I	18 (26.09)	3 (37.50)	0.558
	II	31 (44.93)	2 (25.00)	
	III	16 (23.19)	3 (37.50)	
	IV	4 (5.80)	0 (0.00)	
NC [mm]		400.00 (380.00–440.00)	450.00 (425.00–500.00)	0.011 *
MO [mm]		55.00 (50.00–70.00)	52.50 (50.00–57.50)	0.478
Neck circumference and TMH ratio		7.20 (6.00–9.16)	9.00 (8.29–10.85)	0.037 *

Descriptive data are presented as the number of observations (per cent) or median (Q1–Q3). *: statistically significant; condition of the dentition: 1—full dentition, 2—partial upper dentition, 3—partial lower dentition, and 4—no dentition.

**Table 4:** Multivariable logistic regression analysis for prediction of difficult laryngoscopy.

Independent Variable	Univariable Logistic Regression		Multivariable Logistic Regression	
	OR (±95%CI)	*p*-Value	OR (±95%CI)	*p*-Value
Male gender	0.35 (0.11–1.09)	0.067	0.13 (0.02–0.65)	0.012 *
Sleep apnea	3.21 (1.05–9.84)	0.038	2.21 (0.61–7.94)	0.216
Chin–sternum distance ≤ 175 mm	3.90 (1.25–12.15)	0.017	6.78 (1.42–32.38)	0.014 *
Mouth opening ≤ 60.5 mm	5.88 (1.21–28.66)	0.026	7.35 (1.18–45.87)	0.029 *

*: statistically significant; OR: odds ratio; ±95% CI: ±95% confidence interval.

**Table 5:** Diagnostic potential of selected variables as predictors of difficult laryngoscopy.

	Male Gender	Chin–Sternum Distance [mm]	Opening of the Lips [mm]	Thyromental Height [mm]
AUC (95% CI)	0.612 [0.457–0.768]	0.67 [0.544–0.825]	0.66 [0.510–0.775]	0.53 [0.24–0.64]
*p*-value	0.158	0.010 *	0.035 *	>0.05
SE	0.079	0.072	0.068	0.080
Sensitivity (95% CI)	0.780	0.678	0.424	0.333 [0.133, 0.59]
Specificity (95% CI)	0.444	0.667	0.889	0.729 [0.597, 0.836]
Accuracy (95% CI)	0.701	0.675	0.532	0.636 [0.519, 0.743]
PPV (95% CI)	-	0.387 [0.218, 0.578]	0.320 [0.195, 0.47]	0.273 [0.107, 0.502]
NPV (95% CI)	-	0.87 [0.737, 0.951]	0.926 [0.757, 0.991]	0.782 [0.650, 0.882]

*: statistically significant; AUC: area under ROC curve; 95%CI: 95% confidence interval; SE: standard error.

**Table 6:** Diagnostic potential of OPERA Score as predictor of difficult laryngoscopy.

	OPERA Score	Mallampati Score III/IV	Opening of the Lips [<35 mm]	Thyromental Height [<50 mm]	Neck Circumference [>430 mm]	History of OSA
AUC	0.81	0.54	0.66	0.6	0.71	0.75
*p*-value	0.01 *	>0.05	0.035 *	>0.05	0.04 *	>0.05

*: statistically significant; AUC: area under curve.

## Data Availability

The datasets generated during and/or analyzed during the current study are available from the corresponding author upon reasonable request.

## Abbreviations

The following abbreviations are used in this manuscript:

TMHT	Thyromental Height Test
BMI	Body Mass Index
ASA	American Society of Anesthesiologists
MMT	Modified Mallampati Test
TMD	Thyromental Distance
SMD	Sternomental Distance
NC	Neck Circumference
MO	Mouth Opening
TOF	Train of Four
OSAS	Obstructive Sleep Apnea Syndrome
ROC	Receiver Operating Characteristic
AUC	Area Under the ROC Curve
SE	Standard Error
CI	Confidence Interval
ULBT	Upper Lip Bite Test
